# miR‐221 promotes keratinocyte proliferation and migration by targeting SOCS7 and is regulated by YB‐1

**DOI:** 10.1111/jcmm.17250

**Published:** 2022-02-24

**Authors:** Xiao Feng, Lei Zhang, Wei Feng, Ce Zhang, Tingting Jin, Jingyu Li, Jincai Guo

**Affiliations:** ^1^ Department of Plastic Surgery Zhejiang Provincial People's Hospital People's Hospital of Hangzhou Medical College Hangzhou China

**Keywords:** keratinocytes, miR‐221, SOCS7, wound healing, YB‐1

## Abstract

Proliferation and migration of keratinocytes are vital processes for the successful epithelization specifically after wounding. MiR‐221 has been identified to play a potential role in promoting wound regeneration by inducing blood vessel formation. However, little is known about the role of miR‐221 in the keratinocyte proliferation and migration during wound healing. An in vivo mice wound‐healing model was generated; the expression levels of miR‐221 were assessed by qRT‐PCR and fluorescence in situ hybridization. Initially, we found that miR‐221 was upregulated in the proliferative phase of wound healing. Further, in an in vivo wound‐healing mice model, targeted delivery of miR‐221 mimics accelerated wound healing. Contrastingly, inhibition of miR‐221 delayed healing. Additionally, we observed that overexpression of miR‐221 promoted cell proliferation and migration, while inhibition of miR‐221 had the opposite effects. Moreover, we identified SOCS7 as a direct target of miR‐221 in keratinocytes and overexpression of SOCS7 reversed the effects of miR‐221 in HaCaT keratinocytes. Finally, we identified that YB‐1 regulates the expression of miR‐221 in HaCaT keratinocytes. Overall, our experiments suggest that miR‐221 is regulated by YB‐1 in HaCaT keratinocytes and acts on SOCS7, thereby playing an important role in HaCaT keratinocyte proliferation and migration during wound healing.

## 1 INTRODUCTION

Keratinocytes are the major group of cells in the epidermis that are essential for restoration of the epidermis after an injury *via* a process called a re‐epithelialization.^1^ Re‐epithelialization is characterized by restructuring of the wound by migrating and proliferating keratinocytes to the wound periphery, which allows the formation of new epithelium.^2^ Lack of appropriate healing could lead to long‐term consequences such as acute to chronic pain, infections, along with loss of localized physical functions.^3^ Despite its complexities, relatively less is known about the players affecting such wound healing, specifically the factors that affect keratinocyte migration and proliferation.

MicroRNAs (miRNAs) have recently garnered immense research interest specifically in the field of regeneration.^4^ miRNAs are non‐coding RNA that are of approximately 20 nucleotides long with functions ranging from mRNA translation to regulation of gene expression.^5^ Previously, many miRNAs have been associated with wound healing, specifically associated with inflammation and remodelling.^6^ However, few miRNAs have been associated with re‐epithelization.^7^ A study indicated that miR‐210 induces re‐epithelization by inhibiting translation of ISCU1/2 and E2F3.^8^, ^9^ Further, miR such as miR‐21‐5p,^10^ miR‐132‐3p,^11^ miR‐19b, miR‐20a^12^ and miR‐335‐5p^13^ have all been identified to play key roles in re‐epithelization. One such miRNA which is previously studied for its role in angiogenesis is miR‐221. Specifically, miR‐221‐3p was identified to play a critical role in capillary formation through its regulation of c‐Kit mRNA.^14^ However, no study has illustrated the role of miR‐221 in proliferation and migration of keratinocytes.

Wound healing requires a wide variety or cocktail of cytokines and growth factors; however, their production are tightly regulated by many inhibitors including the SOCS family members. Among the many key players in wound healing, the suppressor of cytokines (SOCS) family members are well known as prognosis predictors for healing related issues.^15^ Increased expression of SOCS1, 2, 5 and 6 have been associated with poor prognosis for wound healing.^15^ Another member of the SOCS family, SOCS7 has been observed to be highly expressed in healing wounds among patients with chronic wounds.^15^ However, this observation need to be explored further to understand its significance in re‐epithelialization. In a study by Rao et al., miR‐221 was identified as a key regulator and suppressor of SOCS7.^16^ SOCS7, which inhibits Janus kinase/signal transducer and activator of the transcription (JAK2‐STAT3),^17^ is widely accepted as a key regulator of proliferation and differentiation. In the absence of SOCS7 regulation, binding of STAT3 and JAK leads to STAT3 phosphorylation and activation, p‐STAT3 then migrates to the nucleus and activates many important cytokines and growth factors required for proliferation.^18^ Hence, regulation of SOCS7 could provide key strategies to improve wound healing. However, the key player that regulates SOCS7 levels thus allowing the appropriate healing of the wounds is still unclear.

In this study, we aim to identify the regulatory role of miR‐221 on the expression of SOCS7 and p‐STAT3. Further, using in vitro and in vivo models, we aim at systematically elucidating the complex mechanism through which miR‐221 is modulated, thus allowing its positive effect on proliferation and migration of keratinocytes during wound healing. To our knowledge, this is the first study to identify the role of miR‐221/SOCS7/p‐STAT3 axis on wound healing.

## 2 MATERIALS AND METHODS

### 2.1 Cell culture

HaCaT keratinocytes were obtained from Procell Life Science&Technology Co., Ltd (Wuhan, China). Further, the cells were cultured in Dulbecco's modified Eagle's medium (DMEM, Life Technologies) with 10% foetal bovine serum (FBS) and 1% penicillin–streptomycin solution (P/S). Cells were cultured at 37℃ in 5% CO_2_.

### 2.2 Quantitative real‐time PCR (qRT‐PCR)

Total RNA was extracted from cells and tissues using Trizol, and the quantity and quality of total RNA were assessed using NanoDrop2000 (Thermo Fisher Scientific). RevertAid First Strand cDNA Synthesis Kit (Thermo Fisher Scientific) was used to reverse transcribe the mRNA to cDNA. Further, relative gene expression was assessed using FastStart Universal SYBR Green Master (Rox) (Roche, Shanghai, China) on real‐time PCR System (ABI 7300, Applied Biosystems). To assess the average expression of the respective genes, 2∧(−△△CT) method was used with normalization performed using GAPDH. Primer sequence of miR‐221‐3p is as follows: Fp‐AACCGGAGCTACATTGTCTGCT; Rp‐ CAGTGCAGGGTCCGAGGT. Normalization of microRNA expression was achieved using U6 snRNA as the internal control.

### 2.3 Western blotting

HaCaT keratinocytes or skin samples after liquid nitrogen grinding and tissue crusher treatment were lysed using ice‐cold lysis buffer containing phenylmethylsulfonyl fluoride protease inhibitors (Beyotime Biotechnology, Shanghai China). After 30 minutes, the sample was centrifuged at 13000 g for 10 mins (4 °C) and the supernatant was quantified for the presence of protein using BCA protein assay (Beyotime). Total protein at the concentration of 30 μg were loaded onto 10% SDS‐PAGE under conditions that are denaturing and migrated. Further, the separated protein was then transferred onto a PVDF membrane (Millipore, Bedford, MA, USA) and blocked with 5% non‐fat dry milk for 30 minutes at room temperature. Further, the membranes were incubated with the respective primary antibodies indicated as below: YB‐1 (1:1000, Santa Cruz Biotechnology, Dallas, TX, USA), SOCS7 (1:1000, Santa Cruz Biotechnology), STAT3 (1:1000, Cell Signaling Technology, Beverly, MA, USA) and p‐STAT3 (1:1000, Cell Signaling Technology) followed by secondary antibodies. Visualization of the blots was achieved through chemiluminescence detection system (Pierce).

### 2.4 Cell transfection

Cells were transfected with either 1 μg/ml mimics of miR‐221 or microRNA mimic negative control, 1 μg/ml miRNA inhibitor (anti–miR‐221) or negative control (anti–miR‐Ctrl) which were obtained from GenePharma.

For SOCS7 overexpression, SOCS7 coding sequence (CDS) was cloned and inserted into pcDNA3.1 plasmid (Invitrogen), and 2 μg pcDNA3.1‐SOCS7‐expressing plasmid was used to transfect cells. To assess the influence of YB‐1, cells were transfected with either 2 μg/ml YB‐1 siRNA (GeneChem, shanghai, china) or with their respective controls. YB‐1 target sequences: 5'‐GTTCCAGTTCAAGGCAGTAAA‐3’, siRNA scrambled: 5'‐GAGCAGCGATATAGTACATCT‐3'. All transfection experiments were carried out using Lipofectamine transfection reagent (Invitrogen).

### 2.5 Cell proliferation (WST‐8) assay

Initially, 2 × 10^3^ HaCaT keratinocytes were seeded onto the 96‐well plate and cultured in DMEM with 10% FBS for 24 h. Then, cells were transfected with miR‐221 mimics and miR‐221 inhibitors, and the proliferation of cells at different transfection times was detected by WST‐8 assay kit (Dojindo, Kumamoto, Japan) according to the manufacturer's instructions using a Microplate reader (BioTek). Briefly, cells were treated with 10 μl of WST‐8 dye and incubated at 37°C for 1 h. Further, the proliferation was assessed by measuring the absorbance at 450 nm.

### 2.6 Cell proliferation Edu assay

HaCaT keratinocytes were seeded onto 24‐well plate and allowed to grow till 50% confluency. Cells were transfected with miR‐221 mimics and miR‐221 inhibitors. 48h after transfection, the medium was replaced with 10 μM 5‐ethynyl‐2´‐deoxyuridine (EdU) for 2 h. Further, the cells were fixed, permeabilized and stained with EdU (BeyoClick^™^ EdU‐488 detection kit, Beyotime). Fluorescence was finally visualized with Olympus BX53 microscope (Olympus).

### 2.7 Cell migration

To assess the cell migration, we performed Transwell migration assay. To achieve this, we used an 8 μm BD Chamber (BD Falcon, Franklin Lakes). Onto the upper chamber of the insert, 1 × 10^5^ keratinocyte cells were seeded, and to the lower chamber, DMEM containing 10% FBS was added. Post 24 h, the cell migrating through the chamber was stained and assessed using a microscope.

### 2.8 Scratch assay

Cells were initially seeded and allowed to grow up to full confluency. A scratch was made on the middle of the dish with 10 μl pipette tip. Further, the cells were continued to culture and imaged at indicated times. The wound areas were measured using Image J analysis, and data are represented as % wound closures.

### 2.9 Luciferase reporter assays

Initially to generate the reporter constructs for the 3'‐UTR assay, we cloned the 3'‐UTR into the psiCHECK2 vector (Promega Corporation), specifically into the XhoI and NotI sites immediately downstream to the Renilla luciferase stop codon. For mutant 3'‐UTRs, the target region was efficiently mutated using site‐directed mutagenesis. Further, the cells were seeded on 96‐well plates and cultured for 24 h and transfection was performed 0.2 µg of the reported construct which were co‐transfected with miR‐221 mimics or inhibitor or negative control with the aid of Lipofectamine 2000 (Invitrogen). Luciferase assays were performed 48 h after transfection according to the manufacturer's instructions (Promega Corporation).

### 2.10 Mice and wound model

C57BL/6J mice (6 weeks old) were initially anaesthetized using 3% isoflurane (Abbott), and the hair on the back was shaved using an electric shaver. Further, after a wash using phosphate buffered saline, full thickness wounds of 1 × 1.5 cm^2^ were created on dorsal skin. On Days 3, 7 and 10, skin tissues from wound edge were harvested. The progression of wound closure was photographed monitored carefully at the indicated time points, and the wound size was analysed using ImageJ. All animal experiments were approved by the institutional review board of Zhejiang Provincial People's Hospital.

### 2.11 Histological analysis

Skin samples were fixed in 4% paraformaldehyde (PFA) and embedded using paraffin. Further, the paraffin moulds were sectioned at 5 μM thickness and stained using haematoxylin and eosin (H&E, Sigma‐Aldrich). Antigen retrieval of the sections stained with Ki‐67 antibody (1:400; Cell Signaling Technology) were performed by heating slides at 95°C for 15 mins in 0.01 M citrate buffer (pH 6.0). The slides were further stained with secondary antibodies and visualized using peroxidase method (Vector Laboratories, Burlingame). Finally, the sections were visualized using bright field imaging.

### 2.12 Fluorescence In situ *hybridization*

Samples were initially embedded onto a paraffin block at sectioned at 6 μM thickness and deparaffinized. Antigen retrieval was performed by boiling in citric acid buffer for 20 min in a water bath. Sections were further incubated in a 37°C humidified chamber and treated with proteinase K for 25 min. Further, the sections were treated with 100 μl of pre‐hybridization buffer and incubated for 1 h at 37°C. Next, the sections were treated with hybridization buffer containing mmu‐mir‐221‐3p probe 5'‐FAM‐GAAACCCAGCAGACAATGTAGCT‐FAM‐3'. Hybridization was performed overnight at 37°C, and the sections were nuclear stained with DAPI. Negative controls were achieved with samples without probe and incubated with hybridization buffer.

### 2.13 Statistical analysis

Two‐tailed Student's *t*‐test was performed to assess the statistical significance of our data sets. One‐way or two‐way ANOVA along with Bonferroni's test was used to assess the significance among multiple groups. All statistical analysis was performed using GraphPad Prism Version 6. Values were considered statistically significant if the *p* < 0.05.

## 3 RESULTS

### 3.1 miR‐221 is upregulated during skin wound healing in mice

**FIGURE 1:**
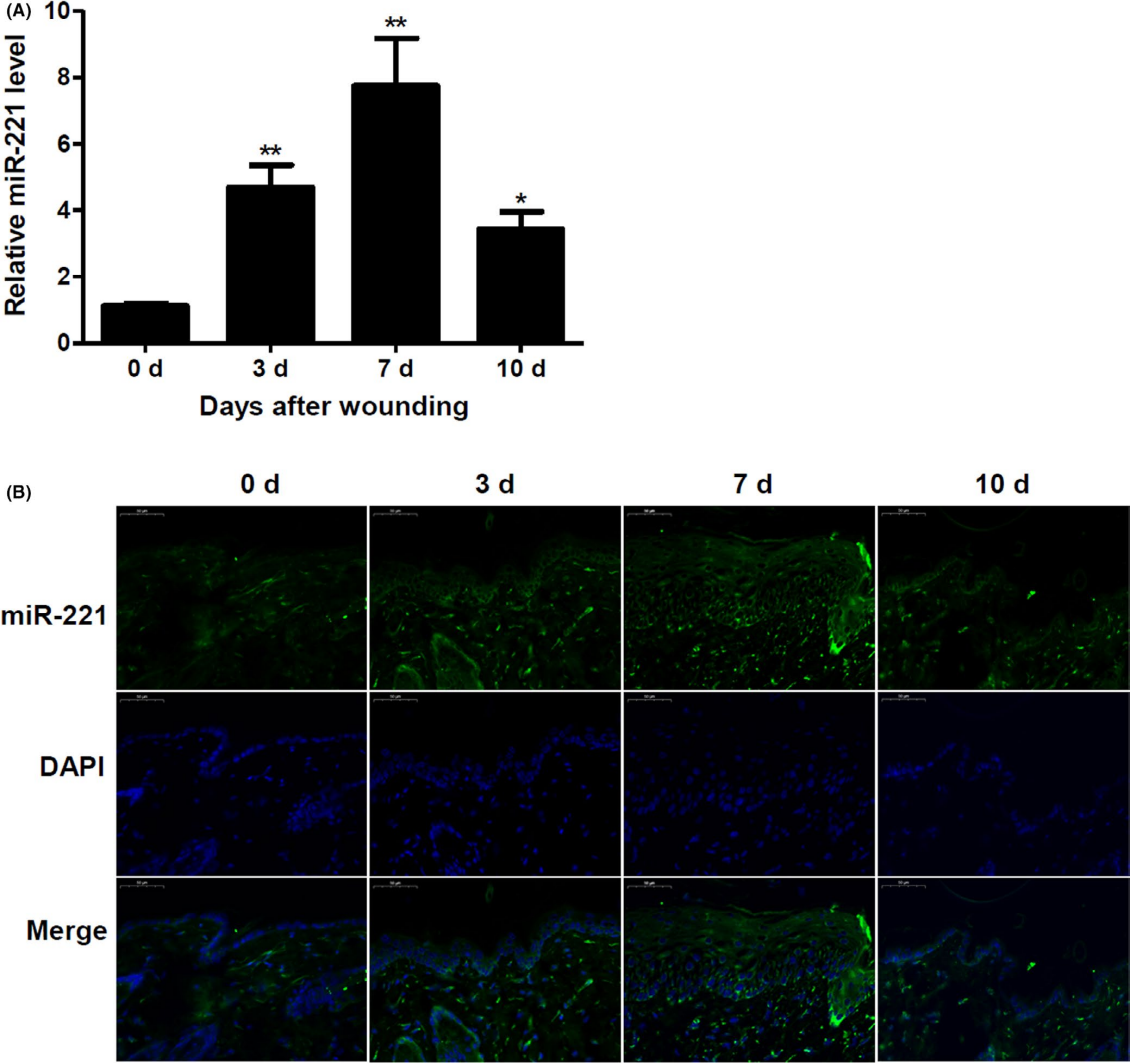
miR‐21 is upregulated during wound healing in mice. (A) The expression of miR‐221 at 3, 7 and 10 days after wounding, relative to unwounded skin were analysed by qRT‐PCR. (*n* = 6). (B) Fluorescence in situ hybridization for miR‐221 performed in mouse macromol wound‐edge skin sections of Days 0, 3, 7 and 10 after wounding. (*n* = 3). Scale bars =50 μm. ^*^ *p* < 0.05, ^**^ *p* < 0.01

To understand the role of miR‐221 during wound healing, we initially generated an in vivo mice wound‐healing model. Further, we assessed the changes in the expression levels of miR‐221 through wound healing from Day 3 to Day 10. Initially, by Day 3, we observed a significant increase in miR‐221 levels (Figure 1A). However, a peak in the levels of miR‐221 was observed at Day 7 followed by a decrease at Day 10 of wound healing (Figure 1A). These results were similarly mimicked when wound‐edge skin were detected by fluorescence in situ hybridization (FISH) for miR‐221, which was mainly expressed in hyperproliferative neo‐epithelium (Figure 1B).

### 3.2 miR‐221 promote skin wound healing

**FIGURE 2:**
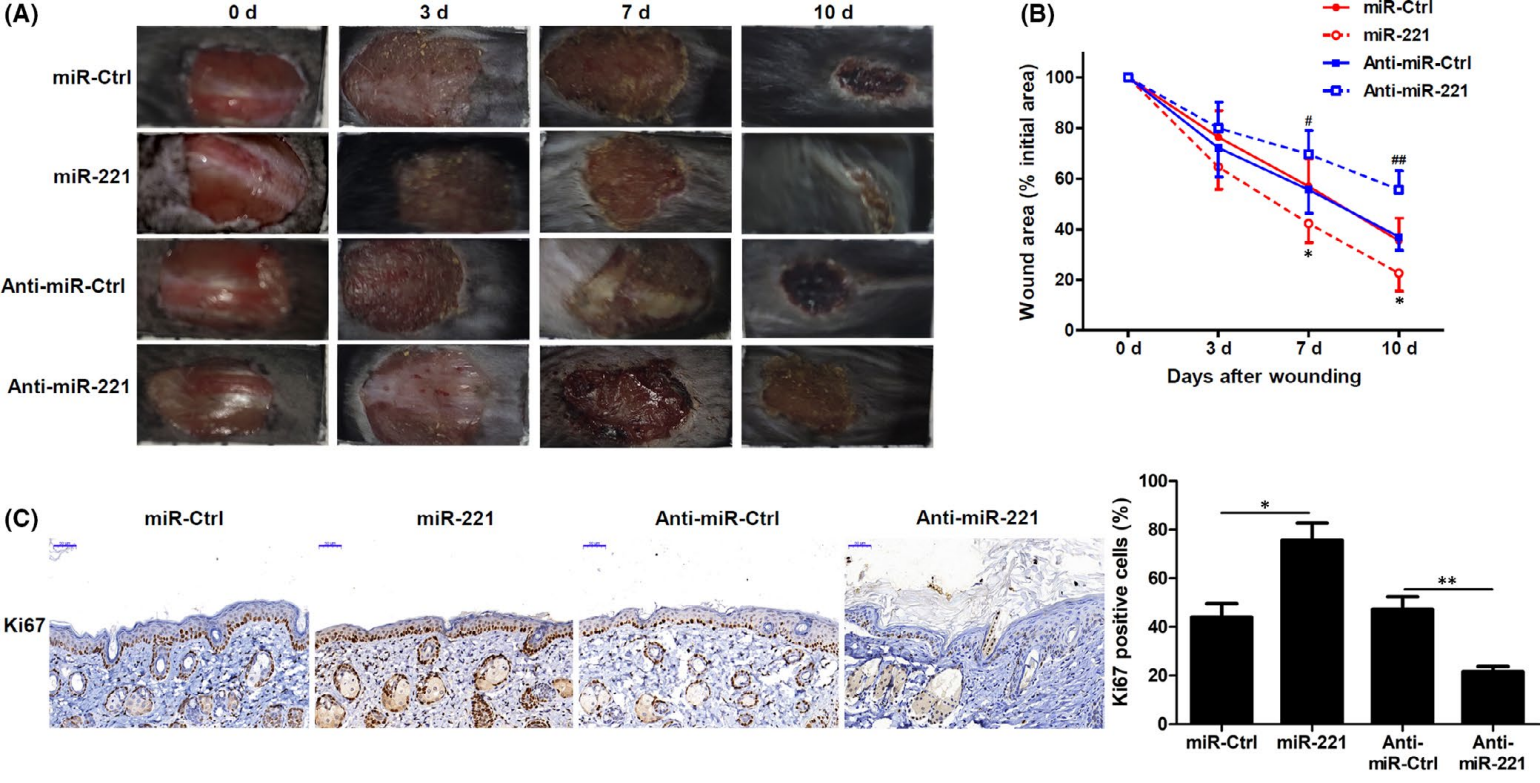
miR‐221 promotes skin wound healing. (A) miR‐221 mimics, anti–miR‐221 or their respective controls were injected intradermally into the wound surface in mice (*n* = 5 for each group) immediately, 4 days and 8 days after wounding. Representative images of wounds on Days 0, 3, 7 and 10 after wounding. (*n* = 6). (B) Quantification of the wound closures was performed, and values are presented as the percentage of the initial wound area size. ^*^ *p* < 0.05 vs miR‐ctrl, ^#^ *p* < 0.05, ^##^ *p* < 0.01 vs Anti‐miR‐ctrl. (*n* = 6). (C) Representative images of Ki‐67 immunohistochemistry of the wound‐edge skin tissues 7 days after injury. (*n* = 3). Scale bars =50 μm. A number of Ki‐67‐positive cells were quantified and presented as percentage of positive cells. ^*^ *p* < 0.05

To further understand the role of miR‐221 on wound healing, we injected miR‐221 mimics or anti‐miR‐221 intradermally onto wound edges at Day 0, Day 4 and Day 8 of the mice model. Imaging of the wounds clearly indicated that use of miR‐221 mimics increased wound healing with significant closure of the wound by Day 10 of wounding, when compared to its control (Figure 2A,B). However, use of anti‐miR‐221 significantly decreased wound healing compared to its control (Figure 2A,B). The data clearly indicated the striking difference between the closure of the wound using miR‐221 mimics and the delayed healing in the anti‐miR‐221 group (Figure 2A,B). Further, using immunostaining, we confirmed that use of miR‐221 mimics significantly increased Ki‐67‐positive proliferating cells, whereas anti‐miR‐221 significantly decreased Ki‐67‐positive cells (Figure 2C). These results clearly indicate that miR‐221 positively promotes wound healing.

### 3.3 miR‐221 drives keratinocyte proliferation and migration

**FIGURE 3:**
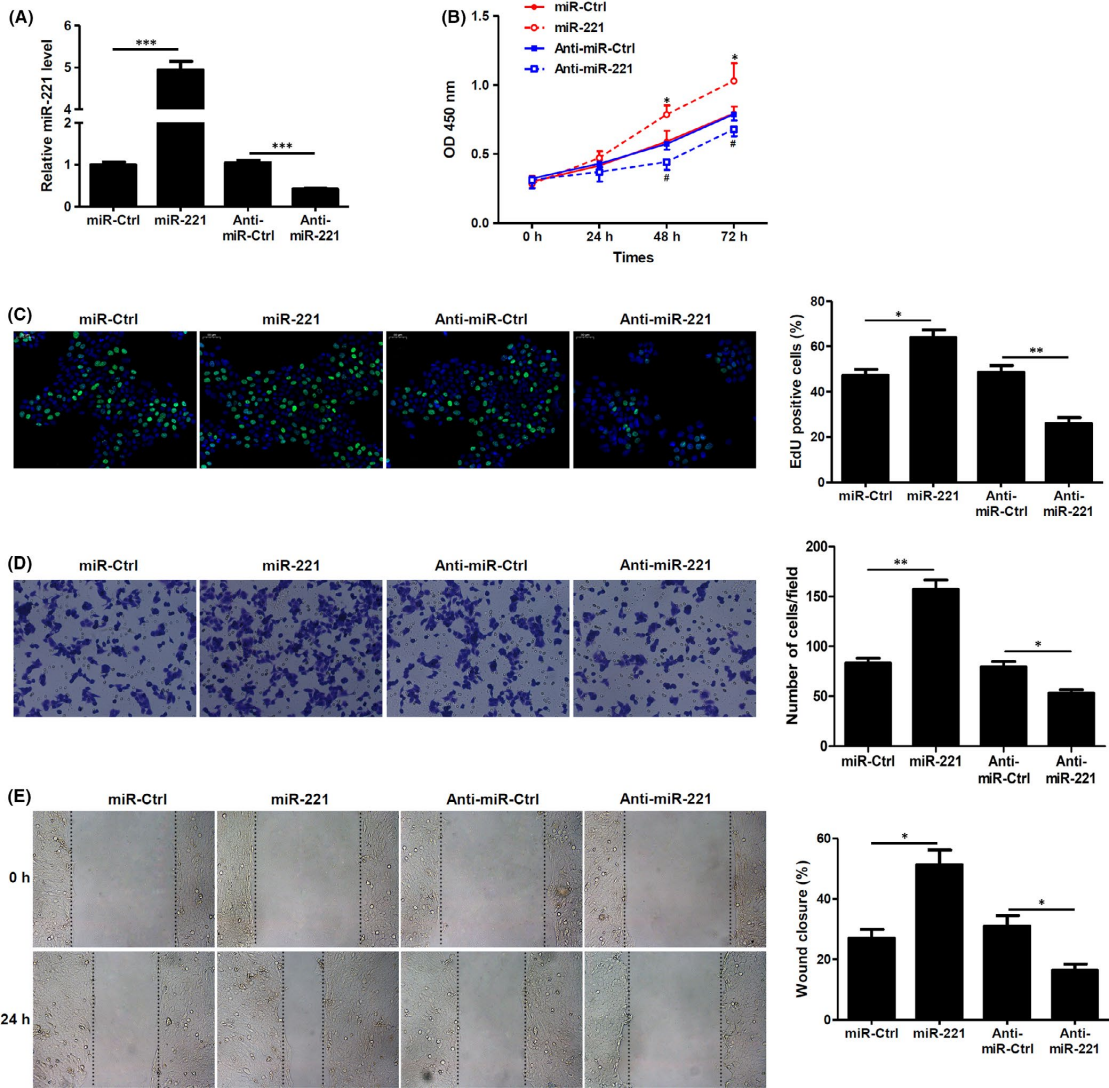
miR‐221 promotes keratinocyte proliferation and migration. (A) miR‐221 mimics, anti–miR‐221 or their respective controls were transfected into HaCaT keratinocytes. Overexpression or silencing of miR‐221 was assessed by measuring the relative expression of miR‐221 after transfection by qRT‐PCR. (*n* = 3). ^***^ *p* < 0.001. (B) Proliferation of HaCaT keratinocytes were assessed using WST‐8 assay. ^*^ *p* < 0.05 vs. miR‐ctrl, ^#^ *p* < 0.05 vs Anti‐miR‐ctrl. (*n* = 3). (C) Cell proliferation was determined by EdU incorporation assay. Percentage of EdU‐positive cells are shown. (*n* = 3). (D) Transwell migration assay of keratinocytes was performed. Representative images are presented here, and the number of cells migrating across the membrane was counted. (*n* = 3). (E) Representative images from the wound scratch assay on HaCaT keratinocytes. Per cent of wound closure was shown. (*n* = 3). ^*^ *p* < 0.05, ^**^ *p* < 0.01

We further performed proliferation and migration assays to assess the effect of miR‐221 on keratinocytes. To achieve this, we used HaCaT keratinocytes as an in vitro model. Initially, we overexpressed or silenced miR‐221 on HaCaT keratinocytes and confirmed miR‐221 expression levels using qRT‐PCR (Figure 3A). Further, we assessed the keratinocyte proliferation using WST‐8 assay. Evidentially, cells overexpressing miR‐221 displayed increased proliferation, whereas cells silenced for miR‐221 showed decreased proliferation, when compared with their respective controls (Figure 3B). We also observed similar results when we performed EdU staining, and it was clear that miR‐221 promoted proliferation of the HaCaT keratinocytes (Figure 3C). Next, we performed transwell migration assay and observed that miR‐221 overexpression increased migration of the cells, when compared to its controls. However, silencing of miR‐221 significantly decreased keratinocyte migration (Figure 3D). Scratch wound assay also indicated that overexpression of miR‐221 increased migration and closure of the wound, whereas silencing decreased the wound closure (Figure 3E). These results indicate that indeed miR‐221 could play a vital role in wound healing by increasing the proliferation and migration of keratinocytes.

### 3.4 SOCS7 is targeted by miR‐221 in keratinocytes

**FIGURE 4:**
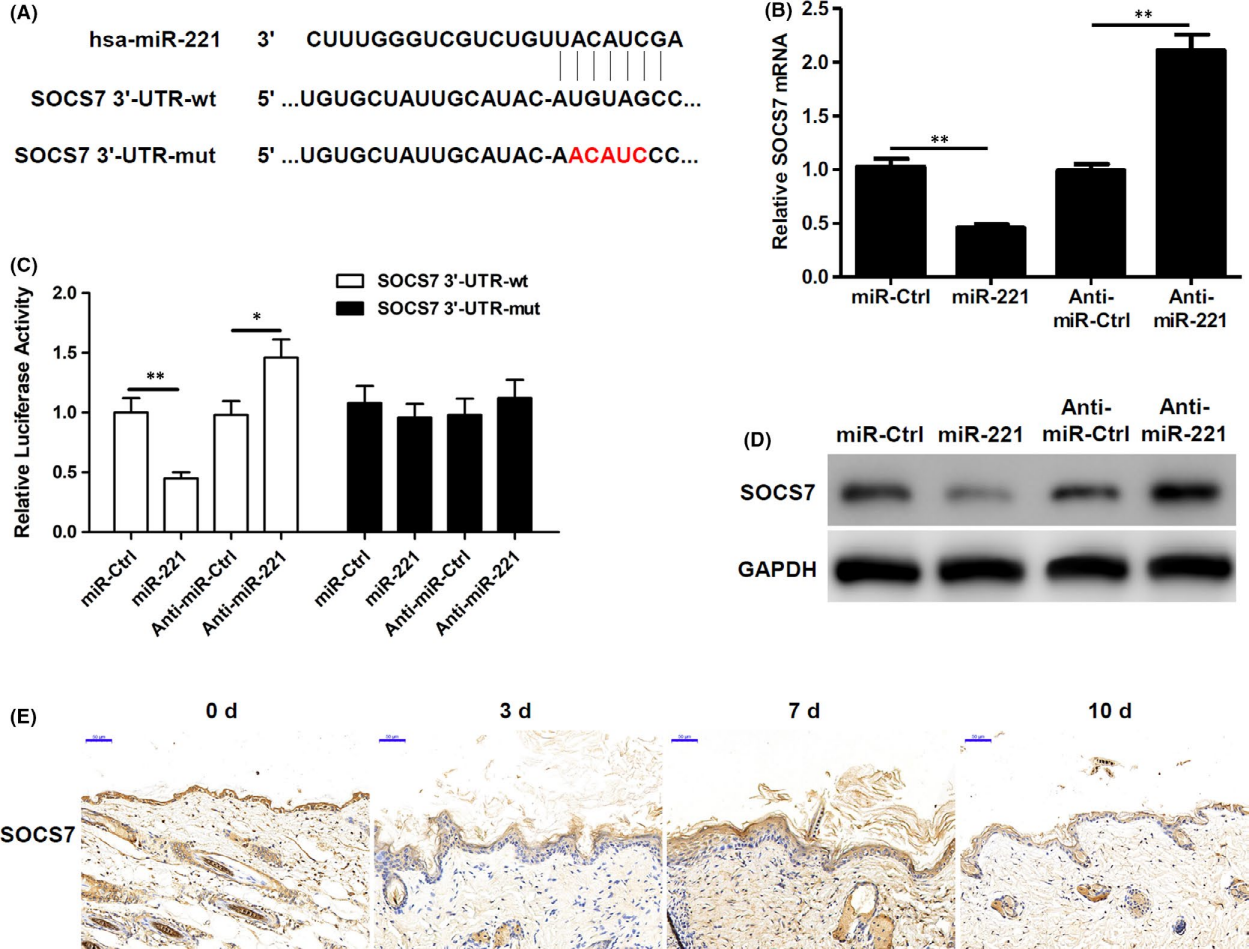
SOCS7 is targeted by miR‐221 in keratinocytes. (A) Mapping of the predicted miR221 binding site in the 3′‐UTR of SOCS7 mRNA: original sequence (green letters) and sequence with miR‐221–mutated binding sites (red letters). (B) Luciferase reporter plasmid containing either the wt or mut SOCS7 3′‐UTR were transfected together with miR‐221 mimics, anti–miR‐221 or their respective controls. Luciferase activity was measured 48 h later. (*n* = 3). (C) qRT‐PCR analysis of SOCS7 mRNA expression in keratinocytes transfected with miR‐221 mimics, anti–miR‐221 or their respective controls for 24 h. (*n* = 3). (D) SOCS7 protein were analysed by Western blotting in keratinocytes transfected with miR‐221 mimics, anti–miR‐221 or their respective controls for 24 h. (*n* = 3). (E) SOCS7 immunohistochemistry in mouse wound‐edge skin sections at Days 0, 3, 7 and 10 after wounding. (*n* = 3). Scale bars =50 μm. ^*^ *p* < 0.05, ^**^ *p* < 0.01

Previously, studies have indicated that SOCS7 is a potential target of miR‐221^19^; hence, we wanted to further assess its role in keratinocytes and wound healing. Initially, we generated luciferase reporter constructs containing either a wildtype (wt) SOCS7 3'‐UTR or a SOCS7 3'‐UTR with a mutation (mut) in the putative miR‐221‐binding site (Figure 4A). When miR‐221 was overexpressed, we observed a significant decrease in luciferase activity in cells with wt SOCS7 3'‐UTR. However, in the cells with mut SOCS7 3'UTR, there was no change in the luciferase activity, compared to its controls (Figure 4B). Further, when cells were silenced for miR‐221, we observed a significant increase in luciferase activity in wt SOCS7 3'‐UTR cells, whereas in cells with mut SOCS7 3'‐UTR, there was no change in luciferase activity (Figure 4B). Further, we also confirmed these observations by checking SOCS7 mRNA and protein levels in the presence or absence of miR‐221 (Figure 4C,D). Evidentially, when miR‐221 was overexpressed, SOCS7 mRNA and protein levels were significantly decreased; however, when miR‐221 was silenced, SOCS7 mRNA and protein levels were significantly increased (Figure 4C,D). These results do indicate that indeed miR‐221 binds to the 3'‐UTR region of SOCS7 and regulates its expression. In addition, we observed a decrease in SOCS7 levels after wounding, which mainly expressed in the inner layers of the neo‐epithelia (Figure 4E).

### 3.5 Overexpression of SOCS7 reverse the effects of miR‐221 on HaCaT keratinocytes

**FIGURE 5:**
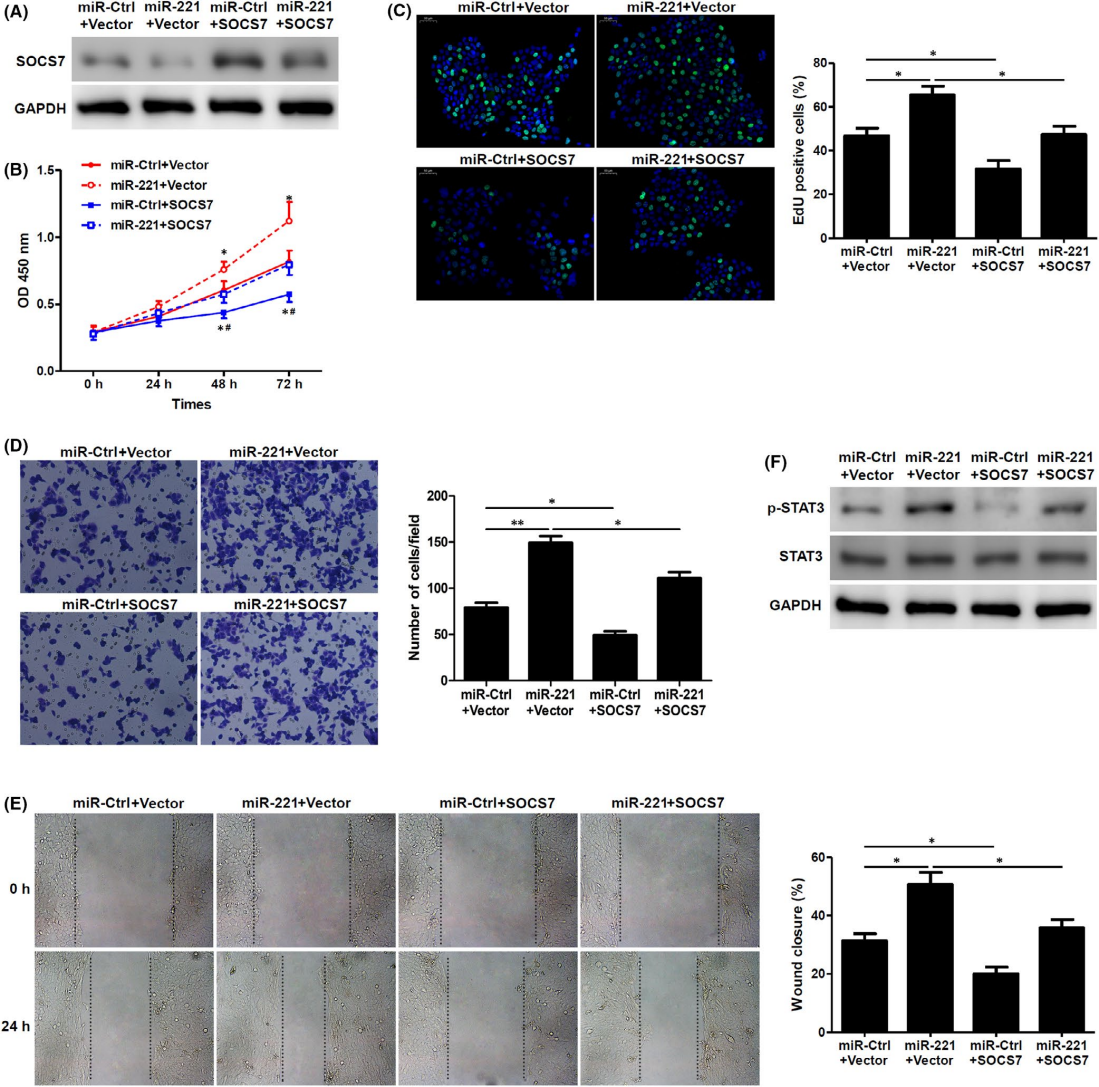
Overexpression of SOCS7 reverse the effects of miR‐221 on HaCaT keratinocytes. (A) SOCS7 protein were analysed by Western blotting in keratinocytes co‐transfected with miR‐221 and SOCS7 overexpression vector or empty vector (Vector) for 24 h. (*n* = 3). (B) Proliferation assay in HaCaT keratinocytes with WST‐8. (*n* = 3). ^*^ *p* < 0.05 vs. miR‐ctrl+Vector, ^#^ *p* < 0.05 vs. miR‐221+Vector. (C) Cell proliferation was determined by EdU incorporation assay. (*n* = 3). Percentage of EdU‐positive cells are shown. (D) Cells migrating through the transwell were assessed using transwell migration assay. (*n* = 3). (E) Per cent of wound closure in the wound scratch assay. (*n* = 3). (F) Phosphorylated and total STAT3 were detected by Western blotting. (*n* = 3). ^*^ *p* < 0.05, ^**^ *p* < 0.01

Next, to further understand the effect of SOCS7 in wound healing, we overexpressed SOCS7 along with miR‐221 in HaCaT keratinocytes. SOCS7 expression was downregulated when miR‐221 was overexpressed in HaCaT keratinocytes. Additionally, when SOCS7 was overexpressed, we reasonably could upregulate SOCS7 protein levels. However, when we simultaneously upregulated SOCS7 and miR‐221, we could observe a significant increase SOCS7 levels compared with the miR‐221 only overexpressed group (Figure 5A). Further, we also performed a WST‐8 proliferation assay and observed that miR‐221 overexpression increased proliferation of HaCaT keratinocytes, whereas overexpression with SOCS7 largely rescue this increase in proliferation induced by miR‐221 overexpression (Figure 5B). These results could be mimicked using an Edu proliferation assay as well (Figure 5C), indicating that indeed overexpression of SOCS7 hindered or decreased the pro‐proliferative effects of miR‐221 in HaCaT keratinocytes. Additionally, we also performed transwell migration and scratch wound assays. Indeed, as we observed previously, miR‐221 overexpression increased migration and wound closure; however, SOCS7 overexpression significantly rescued both migration and wound closure ability induced by miR‐221 overexpression (Figure 5D, E). Since SOCS7 plays a role in STAT pathway, we assessed the levels of phosphorylated STAT3 in HaCaT keratinocytes in the presence or absence of miR‐221. When miR‐221 was overexpressed, phosphorylated STAT3 (pSTAT3) levels significantly increased. However, when SOCS7 was overexpressed, pSTAT3 levels significantly decreased. Expression of SOCS7 in miR‐221 overexpressed cells largely rescued pSTAT3 levels induced by miR‐221 (Figure 5F). Hence, these results indicated that SOCS7 could decrease or reverse the effect of miR‐221 on HaCaT keratinocytes.

### 3.6 MiR‐221 is regulated by YB‐1

**FIGURE 6:**
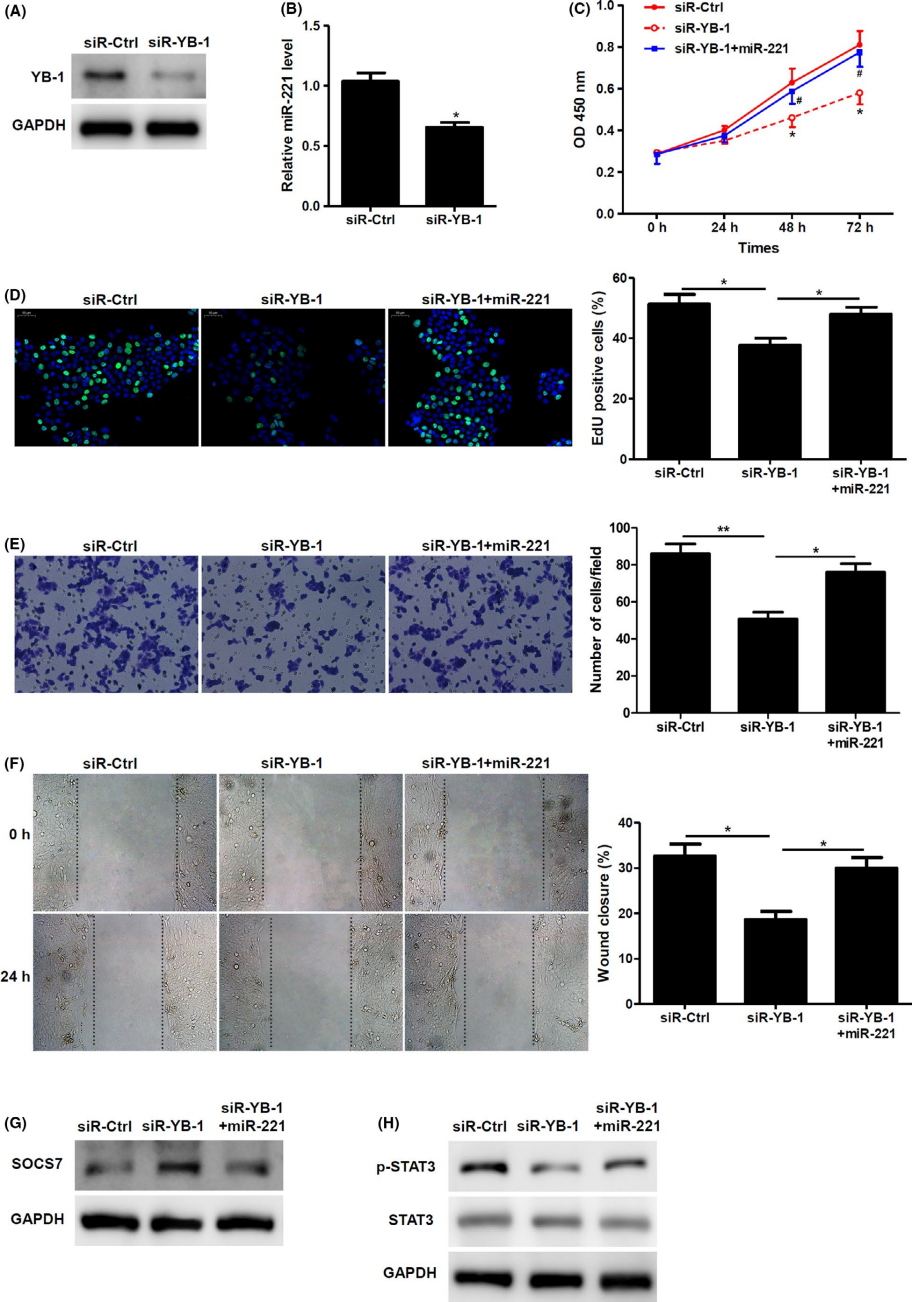
MiR‐221 is directly regulated by YB‐1. (A) YB‐1 protein was analysed by Western blotting in keratinocytes transfected with siRNA‐specific targeted against YB‐1 or scramble control. (*n* = 3). (B) qRT‐PCR was used to assess the relative expression levels of miR‐221 in keratinocytes transfected with YB‐1 siRNA or scramble control. (*n* = 3). (C–F) Keratinocytes were co‐transfected with YB‐1 siRNA or scramble control with miR‐221. (*n* = 3). Proliferation assay in HaCaT keratinocytes with WST‐8 (C). ^*^ *p* < 0.05 vs siR‐ctrl, ^#^ *p* < 0.05 vs. siR‐YB‐1. Cell proliferation was determined by EdU incorporation assay. Percentage of EdU‐positive cells are shown (D). The number of keratinocytes migrating through the membrane in a transwell migration assay was assessed (E). Per cent of wound closure in wound scratch assay is as shown (F). (G) SOCS7 protein were analysed by Western blotting in keratinocytes co‐transfected with YB‐1 siRNA or scramble control with miR‐221. (*n* = 3). (H) Phosphorylated and total STAT3 were detected by Western blotting. (*n* = 3). ^*^ *p* < 0.05, ^**^ *p* < 0.01

**FIGURE 7:**
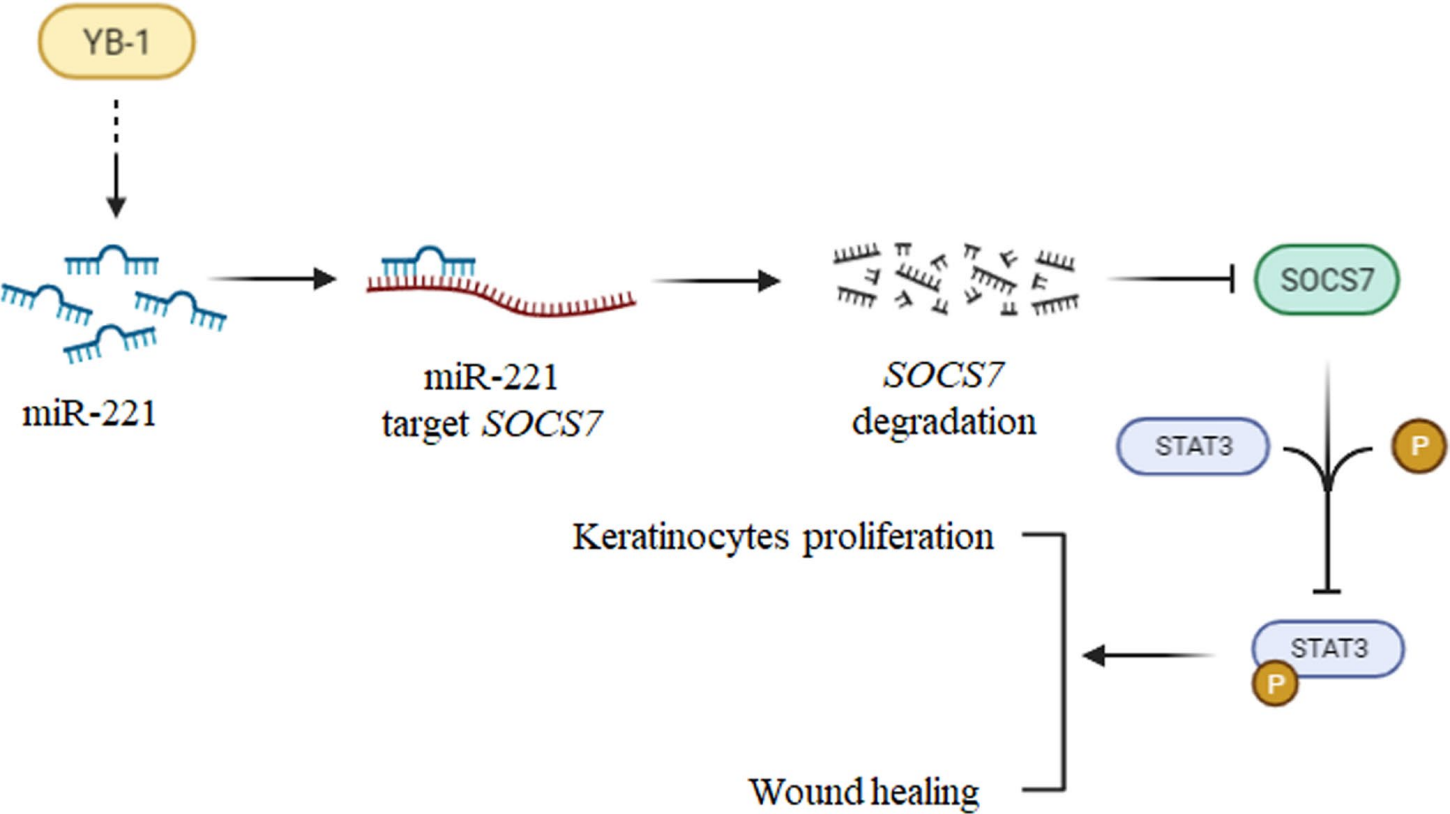
Schematic diagram of miR‐221 targeting SOCS7 to promote keratinocyte proliferation and wound healing. In keratinocytes and during skin wound healing in mice, YB‐1 was able to upregulate miR‐221, which made miR‐221 target to degrade the mRNA of SOCS7, decrease the expression of SOCS7, increase the phosphorylation of STAT3, upregulate keratinocyte cell activity and promote skin wound‐healing progression

Previously, a study has indicated that miR‐221 is upregulated by YB‐1 in glioblastoma.^20^ We wanted to further assess the mechanism behind miR‐221’s effect on wound healing; hence, we checked if YB‐1 also regulated miR‐221 in keratinocytes. Initially, we silenced YB‐1 and confirmed both at the protein that indeed YB‐1 is downregulated (Figure 6A). However, we observed that silencing of YB‐1 significantly downregulated the miR‐221 levels (Figure 6B). Further, when we performed proliferation assay, it was evident that indeed silence of YB‐1 significantly decreased proliferation, whereas upregulation of miR‐221 in these cells could significantly rescue this decrease in proliferation (Figure 6C). We could observe similar results using Edu proliferation assay (Figure 6F). Additionally, we also performed migration and wound closure experiments where we observed that indeed silencing YB‐1 decreased the migration and wound closure, whereas increased miR‐221 in YB‐1 silenced cells reversed the migration phenotype induced by YB‐1 silencing (Figure 6E, F). Further, it was also evident that silencing of YB‐1 increased SOCS7 and decreased p‐STAT3 levels. However, overexpression of miR‐221 in these YB‐1 silenced cells significantly decreased SOCS7 levels and increased pSTAT3 levels (Figure 6G, H), These results do indicate that indeed miR‐221 is critical for proliferation and migration of HaCaT keratinocytes, and miR‐221 is regulated by YB‐1, as shown in Figure 7.

## 4 DISCUSSION

Wound healing is a complex process characterized by orchestrated effort of multiple cells and cytokines, thus involving inflammatory and proliferative phases.^21^ Delayed or inappropriate wound healing could be a consequence of defects in chemokine signalling or in keratinocyte migration and proliferation.^22^ Wound healing can be characterized by three processes, an initial immune response, a keratinocyte migration and proliferation phase, and remodelling phase.^23^ Keratinocyte proliferation during wound healing is considered the most critical process during the wound healing,^24^ and hence, studies have focused on elucidating this process further so as to develop treatment strategies that could enhance wound healing.^25^

miRNAs are known to regulate gene expression due to its interaction with specific mRNA and thus forming a stable mRNA‐miRNA within a RISC complex (RNA‐induced silencing complex).^26^ miRNAs bind to the sequences in the 3’untranslated regions of the mRNA through Watson–Crick pairing.^5^ This association could further lead to degradation or inhibition of the translation of the target mRNA.^5^ Previously, many miRNAs have been associated throughout the process of wound healing.^4^ Similarly, one vital miRNA previously associated with angiogenesis is miR‐221. A study identified that miR‐221 promotes angiogenesis by binding and suppressing the translation of c‐kit mRNA. C‐kit suppression further allows the endothelial cells to form capillaries.^14^ Other studies have indicated that miR‐221‐3p is essential for the pro‐angiogenic effect of atorvastatin pretreated bone marrow mesenchymal stem cell (ATV‐Exos) thus accelerating the repair of skin tissue in diabetes.^27^ Further, miR‐221‐3p–containing extracellular vesicles (EVs) were also identified to promote VSMC proliferation, migration and phenotype switching.^28^ All these studies indicated that miR‐221 indeed played an important role in angiogenesis. In this study, we identified that, indeed, miR‐221 is highly upregulated during wound healing. Further, we also observed that overexpression of miR‐221 significantly improved the wound‐healing capacity in our animal models. Additionally, it was clear from our in vitro models that indeed upregulation of miR‐221 increased proliferation and migration of keratinocytes, whereas silencing did the reverse.

Previously studies have indicated that miR‐221 regulates tumorigenesis in hepatocellular carcinoma by inhibiting SOCS family members.^19^ SOCS family members are relatively well known in the field of regeneration and healing.^15^ Previously, studies have indicated that indeed the SOCS family members suppresses the expression and activation of many cytokines or growth factors, for example SOCS4 and SOCS5 seems to negatively regulate the expression of EGFR (epidermal growth factor receptor), which is upregulated and is essential during the wound‐healing process.^29^ However, SOCS7 downregulates healing through negative regulation of JAK/STAT pathway due to the suppression of STAT3 phosphorylation.^30^, ^31^ STAT3 phosphorylation is important for transcriptional activation of subsequent genes involved in proliferation and migration, and its increased levels have reported to increase proliferation in many types of cancer.^32^, ^33^ In skin, STAT3 activation leads to increased wound healing, keratinocyte migration and follicle development among in vivo models.^34^ In this study, we observed that indeed miR‐221 binds to the 3'‐UTR and suppresses the transcriptional activation of SOCS7, as indicated by the luciferase reporter assay. Use of miR‐mimics significantly downregulated SOCS7 protein levels whereas silencing of miR‐221 significantly upregulated SOCS7. Further, we could observe that SOCS7 downregulation leads to increased phosphorylation of STAT3 and in turn increased proliferation and migration of keratinocytes. Indeed, previous studies had highlighted this key observation that silencing of SOCS7 significantly decreased p‐STAT3 levels and not STAT3 levels, thus indicating that SOCS7 specifically regulates the activation of STAT3.^35^

In this study, we clearly elucidated the key role and regulation of miR‐221/SOCS7/p‐STAT3 axis. However, we were further interested to understand how miR‐221 was indeed regulated. Previously, a study showed that lack of Y‐box‐binding protein 1 (YB‐1) significantly decreased both pre‐miR‐221/222 and mature miR‐221/222 levels.^20^ YB‐1 is an RNA/DNA‐binding protein that is involved in multifaceted regulation of DNA replication, repair, mRNA transcription, stability and even translation.^36^ Recently, YB‐1 has been investigated for its role in binding and processing of miRNA.^20^ In the present study, we identified YB‐1 regulated miR‐221 expression, but whether this occurred at the transcriptional or post‐transcriptional level requires further investigation. Regardless, the YB‐1 and miR‐221 axis allows the regulation of SOCS7, p‐STAT3 and wound healing. In addition, YB‐1 has been previously identified to play a vital role in wound healing.^37^, ^38^, ^39^ In a study by Kwon et al.,^37^ it was observed that YB‐1 is selectively expressed among keratinocyte progenitors and lack of YB‐1 led to destruction of the skin's cytostructural architecture. Interestingly, downregulation of YB‐1 is essential for these progenitors to differentiate, and studies have indicated its role as a protector against stress.^38^ Further, as a stress response protein during injury, YB‐1 crosslinks and actives smooth muscle actin mRNA, thereby allowing activation of myofibroblasts and progressing the wound‐healing process.^39^ Thus, these studies indicate that YB‐1 plays a key role in the proliferation, migration and differentiation of keratinocytes and myofibroblasts during wound healing. Other studies have indicated that YB‐1 acts as a pro‐proliferator and pro‐metastasis regulator in cancer stem cells.^40^, ^41^ This study clearly illustrates that YB‐1 regulates miR‐221 thereby allowing it to regulate SOCS7/p‐STAT3, consequently leading to increased proliferation and migration, and finally contributing to increased wound healing. This study also thus contributes to the identification of key targets for the treatment of defects in wound healing and chronic wounds.

## CONFLICT OF INTEREST

The authors declare that they have no conflict of interest.

## AUTHOR CONTRIBUTIONS

XF performed the experiments, analysed the data and drafted the manuscript. LZ, WF, CZ, TJ and JL performed the experiments and analysed the data. JG planned, coordinated and designed the experiments and edited the manuscript. All authors contributed to the article and approved the submitted version.

## Supporting information

Supplementary Material

## Data Availability

All data generated in the current study are available from the corresponding author on reasonable request.
