# Supplementary figures and images for: miR‐221 promotes keratinocyte proliferation and migration by targeting SOCS7 and is regulated by YB‐1
Source: J Cell Mol Med. 2022 Feb 24;26(8):2299–311. doi: 10.1111/jcmm.17250 (PMC8995440; doi:10.1111/jcmm.17250)

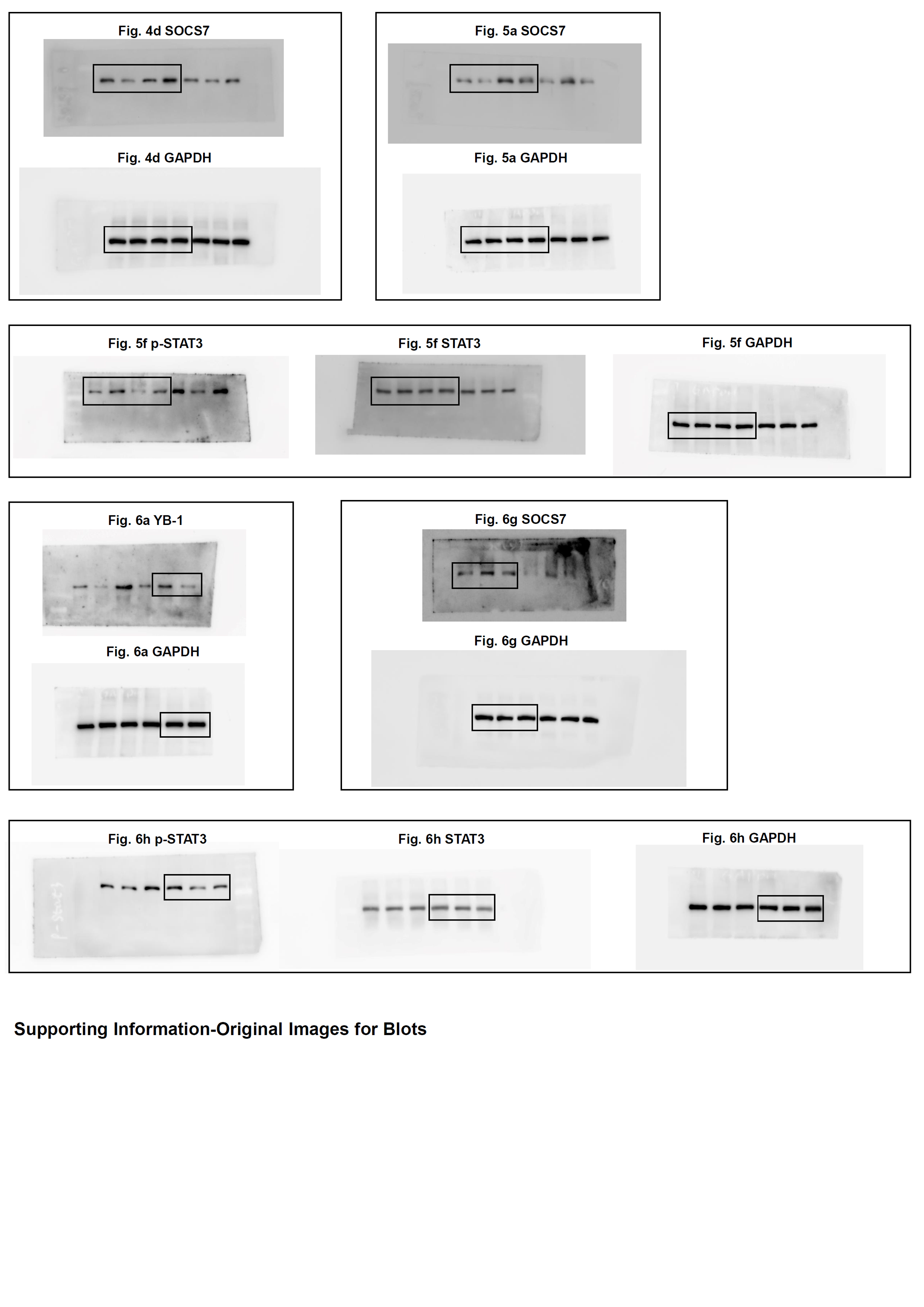

Supplement: Supplementary file 1 — Supplementary Material [file JCMM-26-2299-s001.jpg]
